# *Staphylococcus aureus* Biofilm-Secreted Factors Cause Mucosal Damage, Mast Cell Infiltration, and Goblet Cell Hyperplasia in a Rat Rhinosinusitis Model

**DOI:** 10.3390/ijms25063402

**Published:** 2024-03-17

**Authors:** Ghais Houtak, Roshan Nepal, George Bouras, Gohar Shaghayegh, Catherine Bennett, John Finnie, Kevin Fenix, Alkis James Psaltis, Peter-John Wormald, Sarah Vreugde

**Affiliations:** 1 Adelaide Medical School, Faculty of Health and Medical Sciences, The University of Adelaide, Adelaide, SA 5005, Australia; 2 The Department of Surgery—Otolaryngology-Head and Neck Surgery, The University of Adelaide and the Basil Hetzel Institute for Translational Health Research, Central Adelaide Local Health Network, Adelaide, SA 5000, Australia; 3 Jones Radiology, Eastwood, SA 5063, Australia

**Keywords:** Chronic rhinosinusitis, *Staphylococcus aureus*, biofilm, eosinophilic, IgE

## Abstract

Chronic rhinosinusitis (CRS) is an inflammatory condition of the sinonasal mucosa. Despite being a common health issue, the exact cause of CRS is yet to be understood. However, research suggests that *Staphylococcus aureus*, particularly in its biofilm form, is associated with the disease. This study aimed to investigate the impact of long-term exposure to secreted factors of *Staphylococcus aureus* biofilm (SABSFs), harvested from clinical isolates of non-CRS carrier and CRS patients, on the nasal mucosa in a rat model. Animals were randomised (n = 5/group) to receive daily intranasal instillations of 40 μL (200 μg/μL) SABSFs for 28 days or vehicle control. The sinonasal samples were analysed through histopathology and transcriptome profiling. The results showed that all three intervention groups displayed significant lymphocytic infiltration (*p* ≤ 0.05). However, only the SABSFs collected from the CRSwNP patient caused significant mucosal damage, mast cell infiltration, and goblet cell hyperplasia compared to the control. The transcriptomics results indicated that SABSFs significantly enriched multiple inflammatory pathways and showed distinct transcriptional expression differences between the control group and the SABSFs collected from CRS patients (*p* ≤ 0.05). Additionally, the SABSF challenges induced the expression of IgA and IgG but not IgE. This in vivo study indicates that long-term exposure to SABSFs leads to an inflammatory response in the nasal mucosa with increased severity for *S. aureus* isolated from a CRSwNP patient. Moreover, exposure to SABSFs does not induce local production of IgE.

## 1. Introduction

Chronic rhinosinusitis (CRS) is a chronic inflammatory disease involving the mucosal lining of the nasal passage and paranasal sinuses, affecting 10–15% of the Western population [1,2]. The sinonasal tissue of CRS patients shows a diverse mix of inflammatory cells capable of polarisation towards type 1, 2, or 3 immunity [3]. Phenotypically, chronic rhinosinusitis (CRS) can be classified into subtypes, distinguishing between individuals with nasal polyps (CRSwNP) and those without (CRSsNP). Notably, CRSwNP patients predominantly exhibit a type 2 (T2) immune polarisation characterised by tissue eosinophilia and increased levels of IL-5, IL-44, and IL-13 cytokines. In contrast, most CRSsNP patients do not display inflammatory polarisation and are therefore classified as non-T2 endotypes, often accompanied by increased neutrophil infiltration in the mucosa [4,5]. The pathophysiology of CRS cannot be attributed to a single factor, but rather, multiple host intrinsic and environmental factors are thought to play a role [1]. *S. aureus* is commonly found as a commensal bacterium in the human nares and is highly adaptable to its environment [6,7]. Numerous studies have identified *Staphylococcus aureus* as a potential driver of disease in CRS [8]. Indeed, *S. aureus* is often cultured from the sinuses of CRS patients during the exacerbation of the disease [9]. Furthermore, *S. aureus* induces the secretion of various cytokines such as thymic stromal lymphopoietin (TSLP), IL-33 and IL-22 in nasal mucosa [10,11]. These cytokines activate immune orchestrating cells such as group 2 innate lymphoid cells and T-helper (Th) 2 cells, which in turn drive T2 immunity [12,13]. Another mechanism by which *S. aureus* is thought to be involved in the pathophysiology of CRS is mediated by IgE antibodies targeting *S. aureus* enterotoxins [14,15]. It has been shown that increased IgE contributes to the acute and chronic symptoms of allergic airway disease [16]. Among CRS patients, *S. aureus* enterotoxin-specific IgE is predominantly seen in CRSwNP patients [17]. Multiple lines of research have suggested that the *S. aureus* enterotoxin-specific IgE found in CRS tissue is locally secreted by plasma cells [18,19,20]. *S. aureus* biofilms contain a high amount of virulence factors, many of which are involved in interactions within mucosal environments [21]. In particular, *S. aureus* superantigens have been extensively examined in this context; they are thought to influence the manipulation of airway mucosal immunology towards T2 immune response. This manipulation is mediated by various proteins produced by *S. aureus*, including superantigens, serine-protease-like proteins (Spl), and protein A (SpA), among potentially other factors [22]. Moreover, *S. aureus* biofilms contain proteases which regulate and sustain the biofilm structure, as well as promote dispersal [23]. These proteases, in turn, have been proposed to contribute to the development of a T2 immune response at the mucosal surface [24].

Although the co-culturing of *S. aureus* and respiratory epithelial cells results in the elevation of inflammatory markers in vitro, few studies have investigated the exposure of the nasal epithelium to *S. aureus* biofilm-secreted factors (SABSFs) in vivo. Furthermore, the impact of disease-specific *S. aureus* strains on the nasal mucosa is unclear. Lastly, much uncertainty still exists about the mechanisms that underpin the local secretion of *S. aureus* enterotoxin-specific IgE in the nasal mucosa.

Therefore, this study aimed to elucidate the effect of long-term exposure to secreted factors of *S. aureus* strains harvested from CRS patients and controls grown in biofilm formations.

## 2. Results

**Figure 1:**
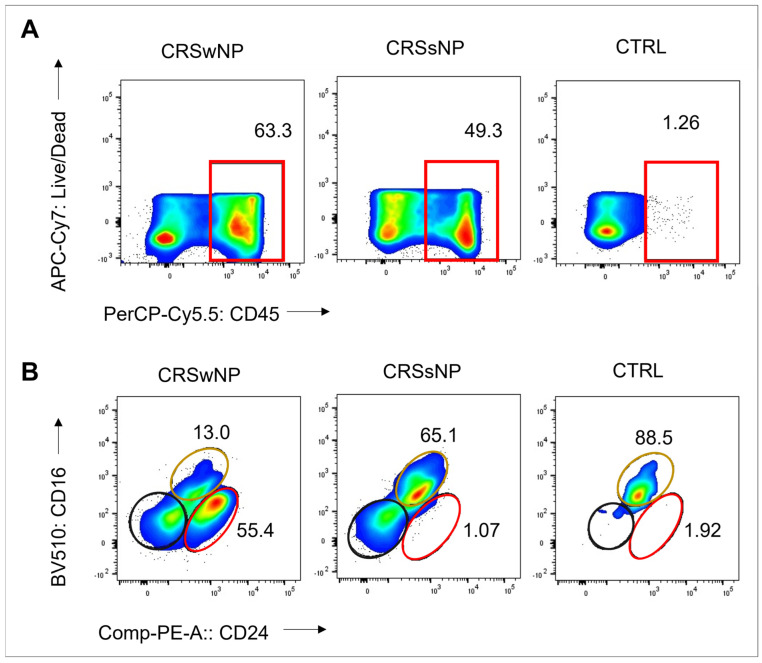
Frequency of CD45+ cells, eosinophils, and neutrophils in sinonasal tissue of CRS patients and non-CRS control. (**A**) Flow cytometry plots show frequency values and cell type gating. The gating of live leukocytes was performed using anti-CD45 PerCP-Cy5.5 antibody and Live/Dead APC-Cy7, following nucleated cell identification via FSC-A versus SSC-A plot and single-cell identification via FSC-A versus FSC-H plot. Eosinophils were identified as CD16- CD24+, neutrophils as CD16+ CD24-, and mast cells as CD16- CD24- from CD45+ and SSC-high granulocytes (Alexa Flour 488-CD14 and SSC-A). (**B**) The frequency of CD45+ cells, eosinophils (represented by the red line), neutrophils (represented by the yellow line), and mast cells (represented by the black line) is shown.

The *S. aureus* strains were isolated from CRS patients with high levels of CD45+ cells: 63% and 49% in the CRSwNP and CRSsNP patients, respectively. In contrast, the non-CRS carrier had a low count of CD45+ cells, indicating non-inflamed mucosal tissue. Eosinophils were the dominant CD45+ cells in the CRSwNP patient (55%), while neutrophils comprised 65% of CD45+ cells in the CRSsNP patient (Figure 1). These findings suggest a T2/eosinophilic endotype for the CRSwNP patient and a non-T2 endotype for the CRSsNP patient.

All rats in the four experimental groups survived the 28-day intranasal challenge with SABSFs without adverse events. The rats showed steady weight gain with increasing age, and no decrease in mean body weight was detected across the groups during the experiments or at the endpoint.

### 2.1. Long-Term SABSF Challenges Induce Multifocal Inflammation

**Figure 2:**
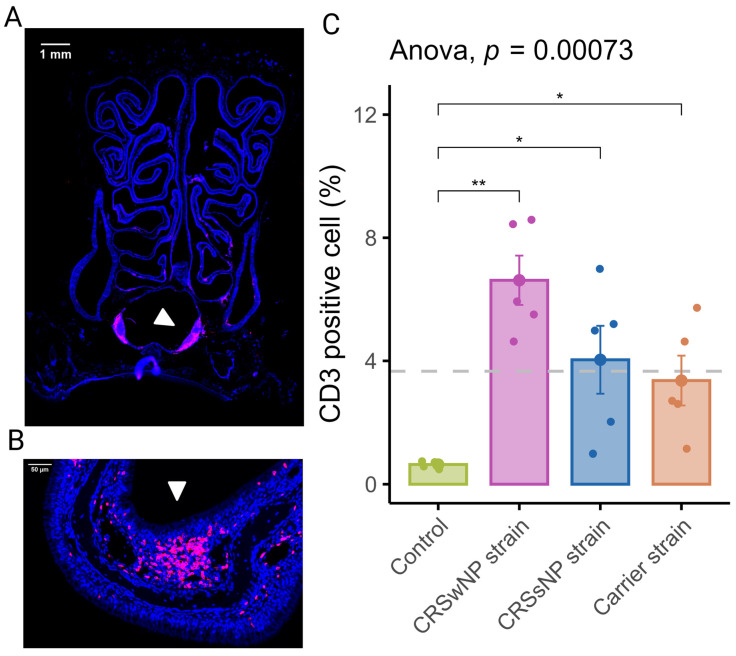
T-lymphocyte infiltration of the nasal mucosa after daily exposure to *S. aureus* biofilm-secreted factors (SABSFs) (vehicle control, SABSF CRSwNP strain, SABSF CRSsNP strain, or SABSF non-CRS carrier strain). (**A**) Representative immunofluorescent whole-slide image of sinonasal coronal sections of a rat’s nose after daily challenge with SABSFs with fluorescent detection of CD3-positive cells (pink, T-cell marker) and 4′,6-diamidino-2-phenylindole (blue, DAPI; nuclear marker). The white arrow indicates the nasal-associated lymphoid tissue (NALT). Scale bar: 1 mm. (**B**) Representative image of the nasal turbinate region with high T-cell infiltration of the olfactory epithelium mucosa located on the endoturbinate (white arrow). Scale bar: 50 µm. (**C**) Percentage of T-cell counts per total cells in mucosa per group. The dashed line represents the mean of all samples. The points represent the individual measurements of the samples (mean of 10 ROI for n = 5 per group). Error bars indicate mean ± s.e.m. * *p* ≤ 0.05, ** *p* ≤ 0.01, one-way ANOVA and pairwise post hoc *t*-test with Benjamini–Hochberg *p*-value adjustment.

In order to assess the inflammation of the nasal mucosa after exposure to disease-specific *S. aureus* strains, we investigated the T-cell infiltration in the mucosa (epithelium and lamina propria) of the septum and turbinates. As shown in Figure 2A, T-cells were scattered within the epithelium, the lamina propria, and the submucosa of the turbinates and the septum. T-cells within the lamina propria and submucosa of the turbinates were often multifocally distributed (Figure 2B). The mean percentage of CD3-positive cells out of total cells in the septum and turbinates was 0.64 (±0.05) for the vehicle control group. The CRSwNP strain group had the highest mean percentage of CD3-positive cells at 6.62% (±0.8). A one-way ANOVA revealed a statistically significant difference between the groups. As shown in Figure 2C, compared to the vehicle control group, T-cell infiltration of the mucosa was significantly increased in all SABSF-treated groups (CRSwNP strain *p* ≤ 0.01, CRSsNP strain *p* ≤ 0.05, carrier strain *p* ≤ 0.05, pairwise post hoc *t*-test with Benjamini–Hochberg *p*-value adjustment).

### 2.2. Long-Term SABSF Challenges of Nasal Mucosa Induce Eosinophilic Infiltration

**Figure 3:**
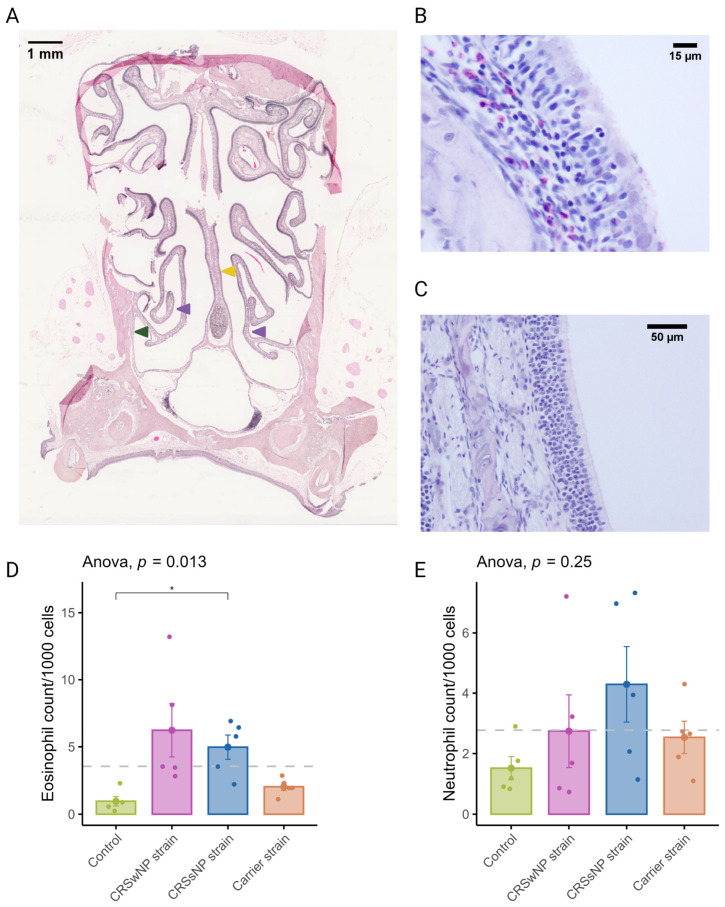
Eosinophil and neutrophil infiltration of the nasal mucosa after daily exposure to *S. aureus* biofilm-secreted factors (SABSFs) (vehicle control, SABSF CRSwNP strain, SABSF CRSsNP strain, or SABSF non-CRS carrier strain). (**A**) Representative histology whole-slide image of sinonasal coronal sections of a rat after 28 days of daily challenge with SABSF stained with H&E. Green arrow indicates lateral nasal wall, yellow arrow indicates nasal septum, and purple arrows indicate turbinates. Scale bar: 1 mm. (**B**) Image of the nasal turbinate region showing a mixed inflammatory infiltrate in the lamina propria, predominantly composed of eosinophils and lymphocytes (the latter also infiltrating the lining epithelium), with fewer neutrophils. Scale bar: 15 µm. (**C**) Representative image of an unaffected area of the nasal turbinates with no inflammatory infiltrate present. Scale bar: 50 µm. (**D**) Quantification of the count (per 1000 cells) of eosinophils in mucosa per group. (**E**) Quantification of the count (per 1000 cells) of neutrophils in mucosa per group. The dashed line represents the mean of all samples. The points represent the individual measurements of the samples (mean of 10 ROI). Error bars indicate mean ± s.e.m. Statistical analysis was performed by comparing groups to control using a one-way ANOVA and pairwise post hoc *t*-test with Benjamini–Hochberg *p*-value adjustment (n = 5 per group). (* *p* ≤ 0.05).

CRSwNP predominantly has a T2 inflammation with a predominantly eosinophilic infiltrate. Eosinophils were quantified in coronal sections to evaluate the potential of SABSFs to induce tissue eosinophilia (Figure 3A). Similarly to T-cells, eosinophilic mucosal infiltration was mainly seen to be multifocally distributed (Figure 3B,C). The mean rate of eosinophils observed in the nasal mucosa of the vehicle control animals was 0.96/1000 cells (±0.35). In the SABSF groups, the mean eosinophil rate was 6.23 (±1.98), 5.00 (±0.90), and 2.04 (±0.29) for the animals stimulated with SABSFs from the CRSwNP strain, CRSsNP strain and carrier strain, respectively. Interestingly, the difference between the vehicle control and CRSsNP strain groups was significant (*p* ≤ 0.05, pairwise post hoc T-test with Benjamini–Hochberg *p*-value adjustment) (Figure 3D). Neutrophils were less frequently observed in the nasal mucosa than eosinophils across all samples (2.77/1000 vs. 3.55/1000). No significant correlation was found between the mean neutrophilic rate of the vehicle control and the SABSF groups (Figure 3E) (ANOVA, *p* = 0.25).

### 2.3. Long-Term SABSF Challenges Induce Mucosal Damage and Goblet Cell Hyperplasia of the Nasal Mucosa

**Figure 4:**
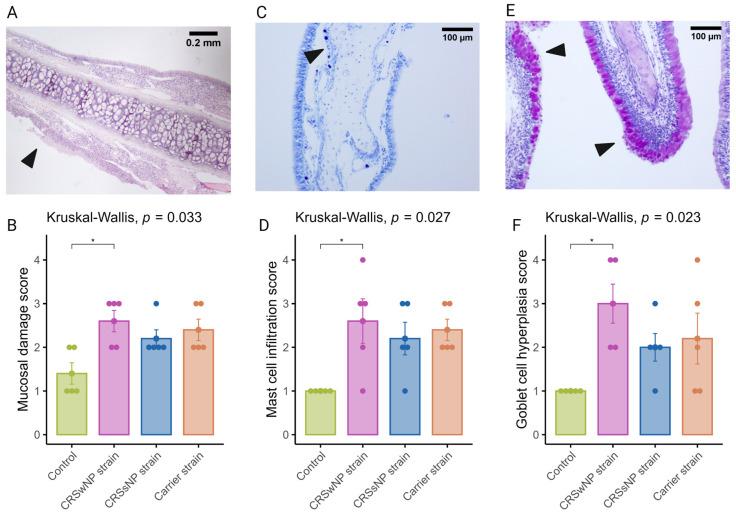
Semi-quantitative histopathologic score assessing mast cell infiltration, mucosal damage, and goblet cell hyperplasia after chronic exposure to *S. aureus* biofilm-secreted factors (SABSF) (vehicle control, SABSF CRSwNP strain, SABSF CRSsNP strain, or SABSF non-CRS carrier strain) for 28 days (scoring scale, Table 1). Mucosal damage was assessed in sections stained with H&E, mast cell infiltration in sections stained with toluidine blue, and goblet hyperplasia in sections with PAS staining. (**A**) In the image of mucosal damage, the black arrow indicates mucosal damage with loss of epithelium and basement membrane. Scale bar: 0.2 mm. (**B**) The mean score of mucosal damage per group. (**C**) Goblet cell hyperplasia (arrows) with proliferation and disorganisation of the respiratory epithelium. Scale bar: 100 µm. (**D**) The mean score of mast cell infiltration per group. (**E**) Image of goblet cell hyperplasia; the black arrows indicate proliferation and disorganisation of the respiratory epithelium with hyperplasia of goblet cells. Scale bar: 100 µm. (**F**) The mean score of goblet cell hyperplasia per group. The points represent the individual measurements of the samples. Error bars indicate mean ± s.e.m. Statistical analysis was performed by comparing groups to control using a Kruskal–Wallis test and pairwise post hoc Dunnet test with Benjamini–Hochberg *p*-value adjustment (n = 5 per group) (* *p* ≤ 0.05).

It is now well established that epithelial damage and barrier dysfunction are involved in the pathophysiology of CRS, and several mechanisms can play a role. One of the primary mechanisms leading to mucosal damage is the local accumulation of IgG, which induces the activation of the classical complement pathway and neutrophils [25]. Another characteristic epithelial change observed in CRS is the increased expression of goblet or secretory cells [26,27]. A semi-quantitative scoring system was used to evaluate mucosal changes in the SABSF-challenged animals (Table 1). After 28 days of daily SABSF intranasal instillation, histopathology examination revealed mucosal damage for all three groups with focal epithelial erosion or ulceration (Figure 4A). Although some epithelial erosion was observed in the vehicle control group, most of the mucosa was intact. The mucosal damage in the CRSwNP-strain group was significantly more compared to the vehicle control group (2.6 ± 0.24 vs. 1.4 ± 0.24 in CRSwNP SABSF-treated animals and vehicle control-treated animals, respectively, *p* ≤ 0.05, pairwise post hoc Dunnet test with Benjamini–Hochberg *p*-value adjustment) (Figure 4B). Furthermore, histopathology revealed regions with proliferation and disorganisation of the respiratory epithelium combined with evidence of goblet cell hyperplasia in all three SABSF groups (Figure 4E). Goblet cell hyperplasia was significantly higher in the CRSwNP strain group compared to the vehicle control group (3 ± 0.45 vs. 1 ± 0, *p* ≤ 0.05 in CRSwNP SABSF-treated animals and vehicle control-treated animals, respectively, pairwise post hoc Dunnet test with Benjamini–Hochberg *p*-value adjustment) (Figure 4F).

### 2.4. Long-Term SABSF Challenges Induce Mastocytosis in the Nasal Mucosa

The current body of research examining the function of mast cells in CRS suggests that they may contribute to the pathophysiology of eosinophilic CRS, as evidenced by the significant increase in membrane-attached IgE-positive mast cells in patients with eosinophilic CRS compared to those with non-eosinophilic CRS [28]. Toluidine blue staining revealed aggregated mast cells in the nasal mucosa of SABSF-challenged rats (Figure 4C). Quantification of mast cells was significantly increased in the CRSwNP strain group compared to the vehicle control group (2.6, ±0.5 vs. 0, ±0 in CRSwNP SABSF-treated animals and vehicle control-treated animals, respectively, *p* ≤ 0.05, pairwise post hoc Dunnet test with Benjamini–Hochberg *p*-value adjustment) (Figure 4D).

### 2.5. Transcriptome Profiling by RNA-Seq Reveals Differential Inflammation between SABSF Groups

**Figure 5:**
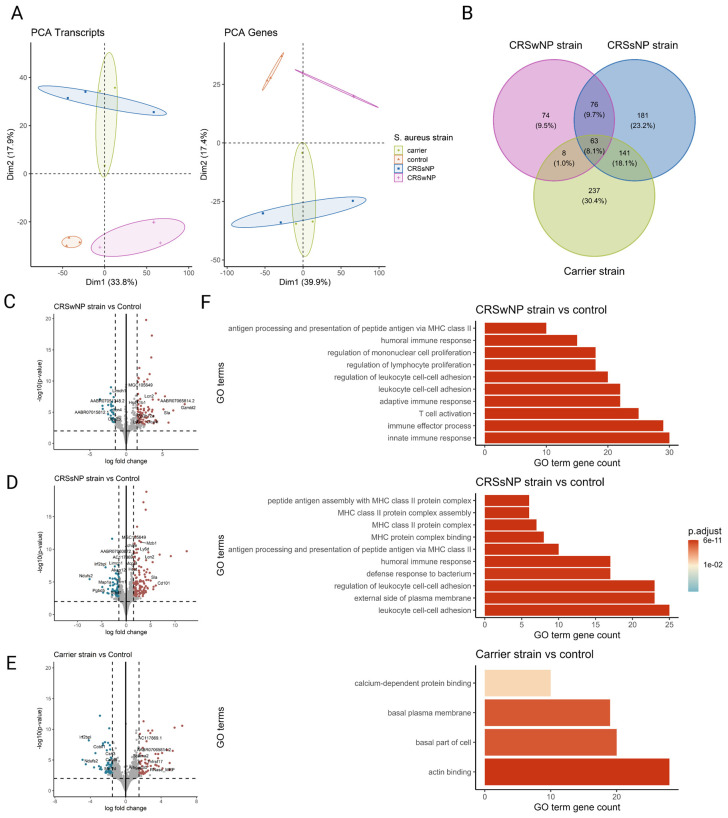
Transcriptome analysis of the nasal mucosal tissue from 12-week-old rats after chronic exposure to *S. aureus* biofilm-secreted factors (SABSFs) for 28 days (vehicle control (n = 3), SABSF CRSwNP strain (n = 3), SABSF CRSsNP strain (n = 3), or SABSF non-CRS carrier strain (n = 3)). (**A**) Principal component analysis based on the top 1000 most variable genes of nasal mucosa transcriptome data on transcript level (left) and gene level (right). Each point represents a sample and is coloured according to the group. The numbers in parentheses on the x and y axes indicate the percentage of variation in the dataset explained by a principal component dimension (PC1 vs. PC2). (**B**) Venn diagram shows the count with the percentage of the total between parentheses of unique differentially expressed genes (DEGs) and overlapping DEGs (adjusted *p*-value ≤ 0.05) between the four groups. (**C**–**E**) Volcano plots of gene expression of all SABSF groups compared to control; significantly upregulated DEGs (adjusted *p* ≤ 0.05) are indicated in red, and significantly downregulated DEGs (adjusted *p* ≤ 0.05) are indicated in blue. Grey dots represent non-DEGs. Data are expressed as Log2 fold change. Vertical dashed lines are set at −1.5 and 1.5. The horizontal dashed line is set at 2. (**F**) Over-representation analysis of Gene Ontology (GO) pathways of DEGs (FDR *p*-value ≤ 0.01). SABSF groups are compared to the control. Only the top 10 most significant pathways are shown per group. PCA, principal component analysis.

To comprehensively study the cellular transcriptional response of the nasal mucosa after SABSF challenges, long-read bulk RNA transcriptomics was performed. The transcriptional response was measured across a total of four conditions and three biological replicates. All comparisons were made to the vehicle control group. A total of 11,258,208; 11,463,508; 7,331,347; and 5,816,055 raw reads were obtained from the control-, CRSwNP strain-, CRSsNP strain-, and carrier strain-treated animals, respectively. Furthermore, 8,971,400; 9,635,611; 6,580,694; and 5,090,261 reads were mapped to the reference genome (Table S1). PCA separated the vehicle control group and SABSF groups on the transcript level (first component 33.8% of the variance, second component 17.9% of the variance) and gene level (first component 39.9% of the variance, second component 17.4% of the variance). However, the expression profiles of the CRSsNP strain group samples were similar to those of the carrier strain group, suggesting a global similarity (Figure 5A). Differential expression analysis grouped by SABSF strain type revealed DEGs between all SABSF groups and vehicle control. The largest number of DEGs was found in the CRSsNP strain group (n = 461, adjusted *p*-value ≤ 0.05) (Figure 5D), closely followed by the carrier strain group (n = 449, adjusted *p*-value ≤ 0.05) (Figure 5E). Fewer DEGs were found in the CRSwNP strain group (n = 221, adjusted *p*-value ≤ 0.05) (Figure 5C). A set of 63 overlapping DEGs were found across all SABSF groups. In particular, a considerable overlap of DEGs between the CRSsNP strain group and the carrier strain group was observed, suggesting a convergent response between these two groups (Figure 5B).

In the CRSsNP strain group, we observed a notable increase in the expression of CD101, a member of the cell-surface immunoglobulin superfamily proteins found on various immune cell subsets including Tregs, memory T-cells, granulocytes, eosinophils, macrophages, monocytes, and DCs. CD101 plays a role in promoting the generation of Tregs and in inhibiting T-cell proliferation and activation [29]. In the CRSwNP strain group, we observed a significant upregulation of the Granzyme B-like 2 gene, encoding a cytotoxic protein known for its role in inducing the death of target cells and microbial pathogens. Originally identified in cytotoxic T-lymphocytes and natural killer cells, Granzyme B-like 2 proteins are pivotal in conferring cytotoxic activities to these immune cells. Extracellular Granzyme B has been shown to degrade the external virulence factors of invading bacterial pathogens, effectively disarming their virulence mechanisms [30]. Furthermore, Granzyme B-like 2 proteins belong to a class of secreted proteases implicated in pathological tissue remodelling [31].

Conversely, one of the most downregulated genes in the CRSwNP strain group is LIMCH1. LIMCH1 is involved in regulating actomyosin activity, facilitating the assembly of actin stress fibres, and stabilising focal adhesions, processes crucial for cell migration. The downregulation of LIMCH1 may indicate a disruption in actin cytoskeleton dynamics and tissue remodelling [32]. Detailed information on significant DEGs is shown in Supplementary Table S2.

Over-representation analysis was performed using GO pathway annotations to identify biological processes, molecular function, and cell compartment genes enriched in the nasal mucosa of SABSF-challenged rats. A total of 99, 39, and 4 GO pathways were enriched for the CRSwNP, CRSsNP, and carrier strain groups, respectively, compared to vehicle control. Despite DEG differences, the over-representation analysis showed strikingly similar enriched pathways for the CRSwNP and CRSsNP strain groups. Among the DEGs, in both the CRSwNP strain group and CRSsNP group, we found enrichment of genes associated with immune effector process, innate immune response, antigen processing and presentation of exogenous antigen, leukocyte cell–cell adhesion, regulation of lymphocyte proliferation, T-cell activation, humoral immune response, adaptive immune response, and regulation of leukocyte cell–cell adhesion, pointing towards a similar biological process in the CRSwNP-strain- and CRSsNP-strain-challenged groups. In contrast, no enriched pathways were implicated in immunity among the DEGs in response to SABSF challenges in the carrier strain group (Figure 5F). Detailed information on GO-enriched pathways is shown in Supplementary Table S3.

**Figure 6:**
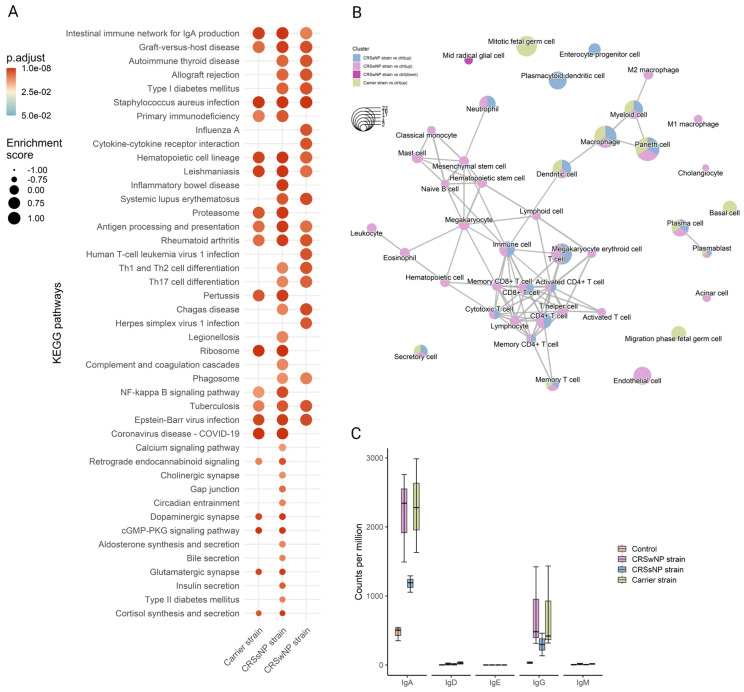
Functional gene expression profile analysis of the nasal mucosal tissue from 12-week-old rats after chronic exposure to *S. aureus* biofilm-secreted factors (SABSF) for 28 days (vehicle control (n = 3), SABSF CRSwNP strain (n = 3), SABSF CRSsNP strain (n = 3), or SABSF non-CRS carrier strain (n = 3)). (**A**) GSEA of pathways in SABSF-challenged nasal mucosa compared to vehicle controls using KEGG annotations. Only gene sets significantly altered (FDR-adjusted *p* ≤ 0.01) compared to the vehicle control group are shown. Columns are ordered by enrichment score. (**B**) Enrichment network map of over-represented cell marker gene sets performed separately for upregulated and downregulated DEGs. Line width and the distance between connected gene sets indicate the similarity of gene sets. Node colour indicates the groups with the significantly enriched term, and node size indicates the count of DEGs count in the gene set. Only significantly enriched cell marker gene sets are shown (FDR-adjusted *p* ≤ 0.01). (**C**) Transcript count (per million) of immunoglobulin heavy chain subtypes in the transcriptome of the nasal mucosal tissue after chronic exposure to *S. aureus* SABSF. Natural killer T-cell, NKT cell; Tumour-associated macrophage, TAM; Tissue-resident memory T-cell, TRM cell; T follicular helper cell, Tfh cell; Innate lymphoid cell, ILC; Exhausted T-cell, Tex cell; Exhausted CD4+ T-cell, CD4+ Tex cell; Exhausted CD8+ T-cell, CD8+ Tex cell.

Next, to detect whole-transcriptome expression perturbations comparing vehicle-control and SABSF-challenged groups, gene set enrichment analysis (GSEA) was performed. We used the KEGG gene sets to determine whether similarly coordinated changes to gene expression were observed along any of the pathways. Nine of the thirty significantly upregulated pathways were found in all SABSF groups (Table S4). Interestingly, the pathway terms were related to infection and immunity, such as “*Staphylococcus aureus* infection”, “Intestinal immune network for IgA production”, “Antigen processing and presentation”, and “Hematopoietic cell lineage”, indicating that immune response pathways were activated. However, the CRSwNP and CRSsNP strain groups included more pathways involved in infection and immunity, such as “Th1 and Th2 cell differentiation”, “Th17 cell differentiation”, “Allograft rejection”, and “Phagosome”, suggesting a more severe immune response (Figure 6A).

Next, functional profile analysis for cell markers was performed to gain insights into the cell types underlying the biological processes of the nasal mucosa. Overwhelmingly, immune cell markers were enriched in the upregulated DEGs of all SABSF-challenged groups (plasma cell, plasmablast, macrophage, dendritic cell, memory T-cell) (Table S5). Notably, non-immune markers such as “secretory cell” were also enriched. Similarly to the KEGG functional analysis, enrichment results for the CRSwNP and CRSsNP strain groups were dominated by cell terms involved in immunity. Interestingly, the CRSwNP strain group had significantly enriched cell markers for eosinophils and mast cells (*p* ≤ 0.05) (Figure 6B).

### 2.6. Long-Term SABSF Challenges Induce Transcription of IgA and IgG

Finally, the antibody production in the nasal mucosa following SABSF challenges was investigated by mapping the IG heavy-chain subtype transcripts. Clear segregation was observed between the four groups’ IG transcripts. Most of the IG transcripts are coded for IgA or IgG subtypes. The CRSwNP and carrier strain groups had the most transcripts coding for IgA, followed by the CRSsNP group. The IgA subtype was predominantly expressed as IgA1 subclass antibodies (Figure S1, expression of immunoglobulin light chain, IgA, and IgG subtypes after chronic exposure to SABSFs). The IgG transcripts showed a similar divergence to IgA between the groups (Figure 6C). Interestingly, most IgG antibodies were expressed as the IgG2A subclass, except for the CRSsNP strain group (Figure S1). Notably, no transcripts were mapped to the IgE heavy chain (Figure 6C).

### 2.7. Virulence Factors Present in the Bacterial Genome

The virulence factors of *S. aureus* have been linked to the activation of different types of immune cells [33]. We investigated these virulence factors in the bacterial genomes and found that the CRSsNP strain contained 54, the CRSwNP strain had 52, and the non-CRS carrier strain had 62 known virulence genes. Notably, the carrier strain was found to carry the superantigen toxic shock syndrome toxin-1 (Figure S2, presence of virulence genes within the genome of clinical isolates). The aureolysin and staphylococcal enterotoxin D virulence factors are notable in the CRSsNP strain, while the collagen binding adhesion gene stands out in the CRSwNP strain.

## 3. Discussion

This study compared the histopathological and transcriptomic response to intranasal challenges with *S. aureus* biofilm-secreted factors derived from strains isolated from CRS patients (both with and without nasal polyps) and a non-CRS carrier strain, using a chronic rodent model.

In chronic infections, *S. aureus* often forms biofilms [34,35], which have been linked independently to disease severity, persistent postoperative symptoms, ongoing mucosal inflammation, and CRS patient infections [36]. Genes exhibit significant differential expression between planktonic and biofilm lifestyles in *S. aureus* [37]. Exoproteins from *S. aureus* biofilms have been shown to induce inflammation and negatively affect the viability and mucosal barrier of nasal epithelial cell cultures [38,39]. Recent studies correlate in vitro *S. aureus* biofilm properties with inflammation levels and the localisation of inflammatory cells in CRS patients [40].

*S. aureus* biofilms are rich in virulence factors, some of which are involved in mucosal interactions [21]. Additionally, these biofilms contain proteases that regulate biofilm structure and facilitate dispersal, potentially contributing to a T2 immune response at the mucosal surface [24]. However, limited research has explored the exposure of nasal epithelium to SABSF in vivo or the specific impact of disease-associated *S. aureus* strains on the nasal mucosa.

The SABSFs used in this study were isolated from mature 48 h *S. aureus* biofilm grown in vitro. Mature biofilms are known for their intricate structures, which undergo dispersal triggered by heightened protease activity. Moreover, the maturation process of biofilms entails dynamic shifts in gene expression and the secretion of extracellular polymeric substances. These factors collectively impact the interaction between bacteria and the host immune system. Our objective in harvesting mature biofilms was to capture a snapshot of the composition of SABSFs, representing the established biofilms within the sinonasal cavity of CRS patients [41,42]. However, since planktonic cells are typically dispersed from such mature biofilms at this growth stage, the SABSFs harvested and used in our experiments likely comprise some exoproteins secreted by *S. aureus* planktonic cells.

Histopathological analysis showed that chronic intranasal challenges with SABSFs induced inflammation, evidenced by significant T-lymphocytic infiltration in the nasal mucosa of the turbinates for all CIs tested compared to vehicle control-treated animals. Interestingly, the inflammation of the mucosal lining was multifocally rather than diffusely distributed. In accordance with the present results, previous studies have also demonstrated an increase in inflammatory cells, such as lymphocytes, neutrophils, eosinophils, and plasma cells, in the subepithelial layer and lamina propria of the nasal mucosa after chronic exposure to *S. aureus* [43,44]. Interestingly, various inflammatory cell type aggregates have also been demonstrated in human nasal tissue and nasal polyps [40]. This study significantly expanded eosinophils after chronic stimulation with the SABSF isolated from a CRSsNP patient. The expansion of eosinophilic cells in animals challenged with the SABSFs from the other two groups showed more variability between animals tested and did not reach statistical significance. Regardless, from the three CIs tested, the SABSF harvested from *S. aureus* isolated from a CRSwNP patient appeared to have the greatest inflammatory propensity, evidenced by the presence of significant mucosal damage, goblet cell hyperplasia, and mast cell infiltration compared to the control. Because SABSF challenges from the three strains were dosed equally, this indicates that the SABSF from *S. aureus* isolated from a CRSwNP patient contained qualitatively different or more factors that can account for this mucosal damage and inflammation. The pathogenicity of *S. aureus* strains is determined indeed by their ability to produce virulence factors. It was therefore somewhat surprising that most virulence factors genes were carried by the carrier strain genome, including Toxic shock syndrome toxin-1, which was not present in the other strains. A potential difference in virulence factor expression profiles between isolates might explain this observation. In the vehicle control group, sporadic CD-3-positive cells were observed in the mucosa, which is not unexpected given the nasal mucosa’s role as an interface for the immune system with the external environment. Similar observations were made regarding neutrophilic and eosinophilic infiltration. However, a few samples from the vehicle control group exhibited minor damage to the nasal mucosa, possibly attributable to the instillation of the vehicle control media. To further pursue the observed difference in inflammation after stimulation with SABSFs, an unbiased approach was employed to investigate the gene expression variation of the inflammatory response of the nasal mucosa. GO, KEGG, and cell marker gene set functional enrichment analysis of the mucosal transcriptome showed a consistent response to the different stimuli across the three SABSF groups, where the primary sources of variation can be attributed to the SABSF group type. The SABSF from *S. aureus* isolated from a CRSwNP patient consistently included more infection and immunity pathways corroborating the previously discussed histopathological findings. In eosinophilic CRS, a hallmark characteristic is the presence of T2 inflammation, marked by a significant infiltration of eosinophils within the nasal tissue. Although our study noted an increase in eosinophilic infiltration, it did not align with the typical pattern observed in eosinophilic CRS. However, we did uncover a notable enrichment of cell markers associated with both eosinophils and mast cells within the CRSwNP *S. aureus* strain group.

While the precise role of mast cells in chronic rhinosinusitis (CRS) and its subtypes remains incompletely understood, their frequent coexistence with eosinophils suggests a potential interaction between these cell types in the context of CRSwNP [45].

Our data reveal a considerable variability in eosinophilic, neutrophilic, and CD-3-positive cell counts within the SABSF groups, contrasting with less variability in the vehicle control group. This discrepancy may stem from the multifocal nature of inflammation induced by SABSFs, posing a potential challenge for repeatability. However, an overarching consistent pattern emerged across all analyses when examining the three SABSF groups collectively.

An initial objective of the project was to assess the production of IgE in the nasal mucosa after chronic exposure to SABSFs, as several studies have shown the presence of *S. aureus*-specific IgE in CRS [18,46,47,48]. The results of this study indicate that exposure to SABSFs did not lead to local production in the nasal mucosa of IgE. Instead, an increase was seen in IgA and IgG production. Ig class switching to IgE is regulated by IL-4, a type 2 immunity cytokine significantly increased in eosinophilic CRS [5,49]. The absence of IgE production might be related to insufficient IL-4 secretion (Th2 cells), as SABSF nasal challenges did not induce a stark T2 immune response. This also accords with the observations of IgG subtypes, which showed that the majority of the IgG was IgG2, as T1 immunity induces the production of IgG2a, whereas type 2 immunity stimulates the expression of IgG1, rendering each isotype an indicator of the underlying immune response [50]. Du et al. presented a noteworthy discovery highlighting that exposure to *S. aureus* lysate, as opposed to inactivated *S. aureus*, led to a significant increase in IgE production in the supernatants of nasal polyp tissues obtained from individuals with CRSwNP [48]. Together, these findings suggest that prolonged *S. aureus* infection/exposure in the nasal cavity might not independently lead to Th cell polarisation and IgE production. It might be that the local *S. aureus*-specific IgE production in CRS occurs in T2 inflammatory states of the nasal tissue preceding or co-occurring with an *S. aureus* infection, leading to the allergic sensitisation to *S. aureus* secreted factors, subsequently perpetuating the chronic allergic inflammatory state in individuals with CRS who are colonised by *S. aureus*. Further studies which consider these variables will need to be undertaken.

The findings of our study should be interpreted in light of several limitations. Firstly, the small sample size presents challenges in detecting significant differences between intervention groups. Increasing the sample size would enhance statistical power and enable more robust comparisons. Additionally, chronic changes, such as airway epithelium remodelling, typically necessitate longer stimulation periods. In contrast, our study utilised a one-month model, potentially limiting its representation of chronic conditions. Moreover, our study only involved three strains of *S. aureus*. Given the significant variability in virulence factors among clonal clusters of *S. aureus* strains, the strains used may not be fully representative of the strains observed in CRS subtypes. Furthermore, although efforts were made to replicate mature biofilms in vivo, it is important to acknowledge that the in vitro growth of biofilms may not precisely replicate the exact conditions present in vivo.

## 4. Materials and Methods

Statement of Ethics: This study complies with all relevant ethical regulations for animal and human research. Ethical approval for animal experimentation was obtained from The University of Adelaide’s Animal Ethics Committee, approval number [M-2019-101], and conducted according to the Australian code for the care and use of animals for scientific purposes [51]. Furthermore, this study protocol was reviewed and approved by The Central Adelaide Local Health Network Human Research Ethics Committee, approval number [HREC/15/TQEH/132]. Written informed consent was obtained from donors to participate in the study.

### 4.1. S. aureus Clinical Isolates

Three clinical isolates (CIs) of *S. aureus* were selected and retrieved from a bacterial biobank consisting of samples stored in 25% glycerol stock at −80 °C, obtained from swabs taken from the sinonasal cavity of subjects (middle meatus) during sinonasal surgery. These swabs underwent in-house processing (purification and re-streaking). Subsequently, all processed swabs were screened for co-colonisation based on colony morphology and colour. The CIs were selected based on the host subject’s disease phenotype and inflammatory pattern in the sinus tissue. Two CIs were from CRS patients: one CRSwNP with a T2 inflammatory endotype and one CRSsNP with a non-T2 endotype [4]. One CI was isolated from a non-CRS control subject with no evidence of sinonasal inflammation based on the absence of CRS symptoms and endoscopic evaluation.

### 4.2. CRS Endotype Confirmation

The inflammatory status of the sinonasal tissue (ethmoid mucosa) derived from the subjects from whom the three *S. aureus* isolates were obtained was assessed using flow cytometry. In brief, fresh sinonasal tissue samples underwent enzymatic digestion with a mixture of 25 mg/mL collagenase D (Roche Diagnostics GmbH, 11088858001, Mannheim, Germany), 10 mg/mL DNase I (Sigma-Aldrich, 11284932001, St. Louis, MO, USA), and Hanks’ Balanced Salt Solution (Thermo Fisher Scientific, 88284, Waltham, MA, USA) for 30 min at 37 °C. The resulting single-cell suspensions were used at a concentration of 4 million cells/mL and stained with Fixable Viability Dye eFluor 780 (BD Bioscience, 565388, San Jose, CA, USA), followed by Fc Block incubation and labelling with fluorochrome-conjugated antibodies (CD45-PerCP-Cy5.5, CD14-Alexa-Flour 488, CD16-BV510, and CD24-PE). Multi-colour flow cytometry was performed using a BD FACS Canto II instrument (BD Bioscience) and FACSDiva software v.6.1.3, with at least 500,000 events collected per sample. Data analysis was performed using FlowJo software v10.8.1.

### 4.3. S. aureus Biofilm-Secreted Factors

SABSFs were used as stimulants. The SABSFs were collected from the supernatant of *S. aureus* after biofilm formation. In brief, the CIs were cultured on Nutrient Agar (Sigma-Aldrich, N7519, USA). Then, single colonies were suspended in 0.9% saline to a 1 McFarland Unit (MFU) turbidity reading. The suspension was diluted 15-fold in Nutrient Broth before incubation in a 6-well microtiter plate (2 mL per well). The inoculated suspension was cultured to induce biofilm formations; the microtiter plates were incubated for 48 h at 37 °C with sheer force on a rotating plate set at 70 rpm (Ratek Instruments, 3D Gyratory Mixer, Boronia, Australia). Following the incubation, the biofilm was dispersed by pipetting (minimum of 10 times), and the bacterial broth culture was collected and centrifuged for 10 min at 1500 relative centrifugal force (rcf) in 4 °C. The supernatants were sterilised through a 0.22 µm acrodisc filter (Pall Corporation, 4612, Port Washinton, NY, USA). The filtered supernatants were concentrated 8-fold using a pre-rinsed 3K MWCO Pierce Protein Concentrator PES (molecular weight cutoff: 3 kDa) (Thermo Fisher Scientific, 88525, USA) by centrifuging at 4000 rcf in 4 °C, according to the manufacturer’s instructions. The protein concentration of the concentrated supernatants was quantified using a NanoOrange Protein Quantitation Kit (Thermo Fisher Scientific, N6666). The concentrated supernatants, or SABSFs, were diluted with Nutrient Broth to a concentration of 200 µg/µL and stored at −80 °C until further use.

### 4.4. Animals and Study Design

**Figure 7:**
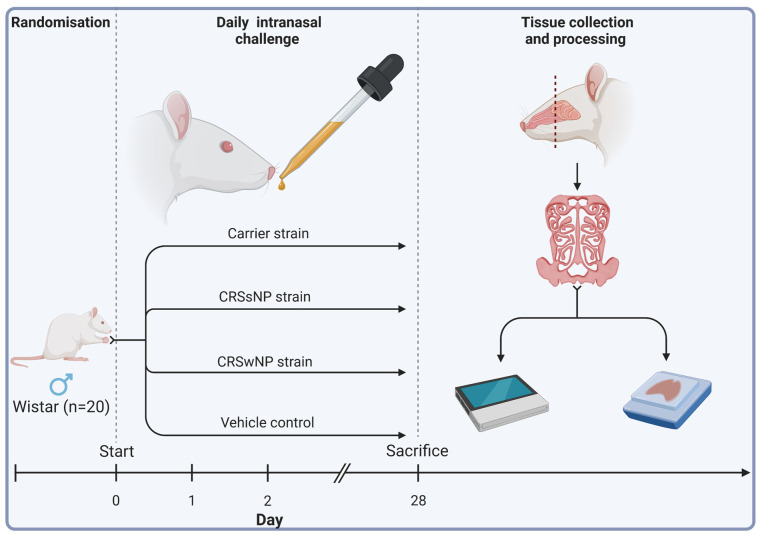
Schematic outline of the experimental design. Twenty male Wistar rats were randomised into four groups (n  =  5 per group). The animals received intranasal challenges of secreted factors of the *S. aureus* biofilms with strains isolated from a CRSwNP patient, a CRSsNP patient, a non-CRS carrier, or the vehicle control solution. Each animal received 40 μL of SABSF (200 µg/µL) intranasally (20 μL in each nare) daily for 28 days, after which the nasal tissue was harvested for histology and transcriptomics. Tissue samples of all animals were processed for histological analysis. Three animals per group were processed for long-read direct cDNA transcriptomics on the Oxford Nanopore Technologies (ONT) platform. Created with biorender.com (accessed on 23 March 2023).

Twenty male Wistar rats (Animal Resources Centre, Perth, Australia) were obtained at 11 to 12 weeks of age. Eleven-week-old male Wistar rats were chosen for their common use in immunological studies and ease of harvesting sinonasal tissue, streamlining experimental procedures and ensuring consistency across studies. Animals were then randomised into four treatment groups receiving either (1) intranasal challenges (nasal instillation) of SABSF harvested from an *S. aureus* strain isolated from a T2 endotype CRSwNP patient, (2) a non-T2 endotype CRSsNP patient, (3) a non-CRS *S. aureus* nasal carrier, or (4) vehicle control solution (n =  5 per group). Using a short induction with isoflurane anaesthetic gas, the SABSF (200 µg/µL) was administered as 40 μL intranasally (20 μL in each nare) daily for 28 days. On day 28, the animals were sacrificed, and the nasal tissue was harvested for histological and transcriptomics analysis (Figure 7).

### 4.5. Tissue Collection

The heads of the animals were collected, and complete transverse sections of the sinonasal structures were cut at 0.5 mm rostral to the anterior margin of the orbit. The rostral section was placed overnight in RNAlater Stabilisation Solution (Thermo Fisher Scientific, AM7020) and stored at −80 °C until RNA extraction, while the caudal sections were fixed in 10% neutral-buffered formalin for 48 h at RT.

### 4.6. Histological Staining

After formalin fixation, the samples were decalcified in 10% ethylenediaminetetracetic acid (EDTA, pH: 7.0) (Sigma-Aldrich, RDD017) solution for two weeks, paraffin-embedded, and 4 µm sections cut and stained with haematoxylin and eosin (H&E). Duplicate sections were stained with periodic acid–Schiff (PAS) and toluidine blue to detect goblet and mast cells. In order to detect T-cell infiltration of mucous membranes, sections were immunostained for CD3, a specific marker for T-cell derivation. For CD3 immunohistochemistry, samples underwent heat-induced antigen retrieval in the Aptum Bio Retriever 2100 (Diagnostic technology, Belrose, Australia) using 10 mM sodium citrate buffer (pH: 6). The sections were incubated overnight with anti-cell marker cluster of differentiation (CD) 3 antibody (1:200, Abcam, ab16669, Cambridge, UK) at 4 °C in a humidified incubation chamber. Afterwards, the sections were incubated in Cy-5 anti-rabbit secondary antibody (1:1000, Jackson ImmunoResearch Labs Inc., AB_2340607, West Grove, PA, USA) for 1 h at 4 °C and counterstained with 4′,6-Diamidino-2-Phenylindole, Dihydrochloride (DAPI) (Thermo Fisher Scientific, D1306). A negative control omitting the primary antibody and a positive control showing the typical pattern of expression of this antigen were run with each batch of slides. No-primary-antibody controls were used to determine background staining intensities.

### 4.7. Histological Analysis

All sections were scanned using the Hamamatsu Photonics Digital Slide Scanner at 40× magnification (NanoZoomer S60, Hamamatsu, Japan). The IHC-IF stained slides were scanned on the Zeiss Axio Scan Z1 Slide Scanner (Carl Zeiss Microscopy, Oberkochen, Germany) at a magnification of 40×. Images were imported into the open-source digital pathology software QuPath v0.3.0 for analysis [52]. Goblet cell hyperplasia, epithelial ulceration, and mast cell infiltration were scored using a semi-quantitative scale (Table 1) [53].

Eosinophilic and neutrophilic infiltration was quantified on H&E-stained sections. In brief, ten randomly selected regions of interest (ROI), each spanning an area of 0.1 mm^2^, were generated within the consistent coronal section for each rat. These regions were strategically positioned to overlay the turbinates, lateral walls of the nasal cavity, or septum mucosa. The total number of cells within each ROI was quantified using cell segmentation based on nuclear detection (the haematoxylin channel). Cell segmentation was performed using the StarDist algorithm incorporated in Qupath [54]. Eosinophils and neutrophils were manually annotated within each ROI. Eosinophils are known for their distinctive features, including large granules and a nucleus typically comprising two non-segmented lobes. Moreover, their granules commonly stain red, facilitating easy differentiation from other granulocytes. Conversely, neutrophils are recognised by their nuclei, which often exhibit multiple lobes per cell (2–5), and their cytoplasm, which is typically faintly visible.

T-lymphocytic infiltration was quantified in a semi-automated workflow on anti-CD3 stained sections. Ten random ROIs with an area of 0.2 mm^2^ were created, overlaying the turbinates, lateral borders of the nasal cavity, or septum mucosa. The total number of cells within the ROI was quantified based on nuclear detection (the DAPI channel) using the StarDist algorithm. Cells with Cy-5 signals above the set threshold (12,500) were classified as CD3-positive T-lymphocytes. The samples were de-identified after the analysis to maintain experimental blinding of the groups.

### 4.8. RNA Extraction

The tissue samples stored in RNAlater Stabilisation Solution (Thermo Fisher Scientific, AM7020) were dissected, and the mucosal layer of the sinonasal cavity was collected for further processing. RNA was extracted from the mucosal tissue using the RNeasy kit (Qiagen, 74104, Hilden, Germany) according to the manufacturer’s instructions. The RNA integrity number (RIN) was analysed for each sample using the TapeStation System (Agilent, model 4200, Santa Clara, CA, USA). The purity of RNA was assessed using the NanoDrop (Thermo Scientific, model ND-1000). The RNA concentration was quantified using the Qubit RNA High Sensitivity kit (Thermo Fisher Scientific, Q32852). After quality control, ribosomal RNA was removed from the extracted samples using the RiboMinus Eukaryote System v2 kit (Thermo Fisher Scientific, A15026) and concentrated with the RiboMinus Concentration Module kit (Thermo Fisher Scientific, K155005).

### 4.9. Library Preparation and Sequencing

Long-read transcriptomics was performed on RNA extracts using the direct cDNA sequencing kit (Oxford Nanopore Technologies, SQK-DCS109, Oxford, UK) according to the manufacturer’s recommendations with the following minor modification: the protocol was started with 200 ng poly(A)-enriched RNA. In short, after first-strand cDNAs were synthesised using the Maxima H Minus Reverse Transcriptase (Thermo Fisher Scientific, EP0752), the RNA was degraded with RNase Cocktail Enzyme Mix (Thermo Fisher Scientific, AM2286) and second-strand cDNAs were synthesised using the LongAmp Taq 2X Master Mix (New England Biolabs, M0287L, Ipswich, MA, USA). The double-stranded cDNAs were end-repaired using the NEBNext Ultra II End Repair/dA-Tailing Module (New England Biolabs, E7546S). Sequencing adapters were ligated to the end-repaired strands using Blunt/TA Ligase Master Mix (New England Biolabs, M0367L). The cDNA libraries were purified after each enzymatic reaction using AMPure XP beads (Beckman Coulter, A63882, Brea, CA, USA). All the samples were loaded on a SpotON flow cell (Oxford Nanopore Technologies, R9.4.1) and sequenced on a MinION (Oxford Nanopore Technologies, Mk1C).

### 4.10. Transcriptomic Quantification

FASTQ base-calling was conducted with Guppy v 6.2.11 super accuracy mode using the ‘dna_r9.4.1_450bps_sup.cfg’ configuration (Oxford Nanopore Technology, Oxford UK). For each sample, all FASTQs were aggregated and run through a customised open-source Snakemake [55] and Snaketool [56] pipeline giantpandrna that can be accessed at https://github.com/gbouras13/giantpandrna (accessed on 23 March 2023). Input FASTQs were aligned with Minimap2 v 2.24 [57] specifying ‘minimap2 -ax splice’ to the Ensembl *Rattus norvegicus* release 108 top-level assembly and gtf file downloaded using the giantpandrna install command [58]. The resulting bam files were sorted using SAMtools v 1.16.1 [59]. These sorted bam files were input for transcriptome discovery and quantification using Bambu v3.0.0 [60].

### 4.11. Immunoglobulin Quantification

Immunoglobulin isotypes were mapped using a custom Python program called NanoReceptor (https://github.com/gbouras13/NanoReceptor, (accessed on 21 January 2023)). Briefly, input transcripts were mapped to the IMGT database [61] using Minimap2, filtered for mapped reads only using SAMtools, and parsed to output Counts per Million transcript values for each Immunoglobulin isotype.

### 4.12. Bioinformatics of Transcriptomics

All subsequent analyses were performed in R v 4.2.0 [62]. Differential gene expression analysis was conducted using the DESeq2 package v 1.38.3 from Bioconductor [63]. The differentially expressed genes (DEGs) significance threshold was set at Benjamini–Hochberg adjusted *p*-value ≤ 0.05. Functions from the tidyverse collection of R packages v 1.3.2 were incorporated into the analysis and visualisation [64]. Pathway enrichment analysis was performed using the clusterProfiler 4.6.0 package [65]. The functional enrichment analysis included the terms Gene Ontology (GO) [66], Kyoto Encyclopedia of Genes and Genomes (KEGG) [67], and CellMarker 2.0 databases [68]. For GO over-representation analyses, all genes expressed in our dataset (n = 12,878) were used as background. The GOSemSim R package v 2.24.0 was used to reduce redundancy among enriched GO terms, with a threshold of 0.7 [69]. Only homolog Ensembl genes of *Homo sapiens* and *Rattus norvegicus* with one-to-one orthologue correspondence were considered for the cell markers. The up- and downregulated genes for each cluster were separately analysed. All genes expressed in our dataset (n = 10,384) were used as background. For the KEGG gene set enrichment (GSEA), pre-ranked GSEA was run on the list of genes, sorted by their log fold changes. The Benjamini–Hochberg method was used for *p*-value adjustment for all functional enrichment analyses, accounting for multiple testing. Only terms with a false discovery rate (FDR) ≤0.01 were considered significant.

### 4.13. Bacterial Genome Sequencing

Whole-genome sequencing of the *S. aureus* clinical isolates was performed at a commercial sequencing facility (SA Pathology, Adelaide, SA, Australia) as previously described by Shaghayegh et al. [40]. In short, the NextSeq 550 platform and the NextSeq 500/550 Mid-Output kit v2.5 (Illumina Inc., San Diego, CA, USA) were used. Genomic DNA was isolated using the NucleoSpin Microbial DNA kit (Machery-Nagel GmbH and Co. KG, Duren, Germany). A modified protocol of the Nextera XT DNA library preparation kit (Illumina Inc.) was employed to develop sequencing libraries. Fragmentation of the genomic DNA and subsequent amplification of the Nextera XT indices to the DNA fragments were performed through a low-cycle PCR reaction. After manual purification and normalisation of the amplicon library, 150 bp reads were generated by sequencing. Using ABRicate [70], version 1.0.1, all isolate contigs were screened via Virulence Factor Database [71] to detect virulence genes.

### 4.14. Statistics

Statistical analysis was performed in R v4.2.1 [62], with data expressed as mean ± standard error of the mean (s.e.m). The histopathological data were compared between groups using a one-way ANOVA test with a post hoc *t*-test for lymphocyte and eosinophil count and a Kruskal–Wallis test with a post hoc Dunnet test for the semi-quantitative scoring. The statistical significance threshold was set at *p* ≤ 0.05, and *p*-values were corrected for multiple comparisons using the Benjamini–Hochberg method.

## Figures and Tables

**Table 1:** Semi-quantitative histopathologic score.

**Score**	**1**	**2**	**3**	**4**
**Mast cell infiltration**	Less than 5 non-clustered mast cells were observed in turbinates, lateral nasal walls, or septum mucosa.	5 to 15 non-clustered mast cells were observed within turbinates, lateral walls, or septum mucosa.	Less than 3 clusters (5 cells) of the mast were observed within the mucosa of turbinates, lateral walls, or septum.	More than 3 clusters of mast cells were observed within the mucosa of turbinates, lateral walls, or septum.
**Score**	**1**	**2**	**3**	**4**
**Mucosal damage**	Less than 3 regions of erosion, ulceration, or necrosis were observed within turbinates, lateral nasal walls, or septum mucosa.	3–6 areas showed exfoliation of the superficial epithelium within the mucosa of turbinates, lateral wall, or septum.	More than 6 regions were observed with epithelial erosion, or less than 6 regions with total loss of epithelium and basement membrane with exposure of the underlying submucosa were observed.	More than 6 regions with complete loss of epithelium and basement membrane were observed, accompanied by exposure of the underlying submucosa and necrosis.
**Score**	**1**	**2**	**3**	**4**
**Goblet cell hyperplasia**	3 or fewer regions were observed with goblet cell hypertropia or hyperplasia within turbinates, lateral nasal walls, or septum mucosa.	3–6 areas with regions were observed with increased numbers of goblet cells and pseudocrypt formation within the mucosa of turbinates, lateral walls, or septum.	More than 6 regions were observed with increased numbers of goblet cells with pseudocrypt formation within the mucosa of turbinates, lateral walls, or septum.	Increased cellularity and disorganisation of the respiratory epithelium were observed, accompanied by hyperplasia of goblet cells within the mucosa of turbinates, lateral wall, or septum.

## Data Availability

The transcriptomics dataset from long-read sequencing is publicly accessible in the Sequence Read Archive (SRA) under accession number PRJNA910244.

## Supplementary Materials

The supporting information can be downloaded at: https://www.mdpi.com/article/10.3390/ijms25063402/s1.

## Abbreviations

CRS	chronic rhinosinusitis
CRSsNP	CRS without nasal polyps
CRSwNP	CRS with nasal polyps
DEG	differentially expressed genes
GSEA	gene set enrichment
H&E	haematoxylin and eosin
IHC-IF	immunohistochemistry staining using immunofluorescence detection
IL	interleukin
MFUs	McFarland Units
NALT	nasal-associated lymphoid tissue
PAS	periodic acid–Schiff
Rcf	relative centrifugal force
RIN	RNA integrity number
ROI	regions of interest
s.e.m	standard error of the mean
SABSF	*S. aureus* biofilm-secreted factors
Th	T-helper
TSLP	thymic stromal lymphopoietin
T2	type 2
