# Supplementary material for: Staphylococcus aureus Biofilm-Secreted Factors Cause Mucosal Damage, Mast Cell Infiltration, and Goblet Cell Hyperplasia in a Rat Rhinosinusitis Model
Source: Int J Mol Sci. 2024 Mar 17;25(6):3402. doi: 10.3390/ijms25063402 (PMC10970533; doi:10.3390/ijms25063402)
Supplement: Supplementary file 1 [file ijms-25-03402-s001.zip › ijms-2844931-Figures and Table S1.pdf]

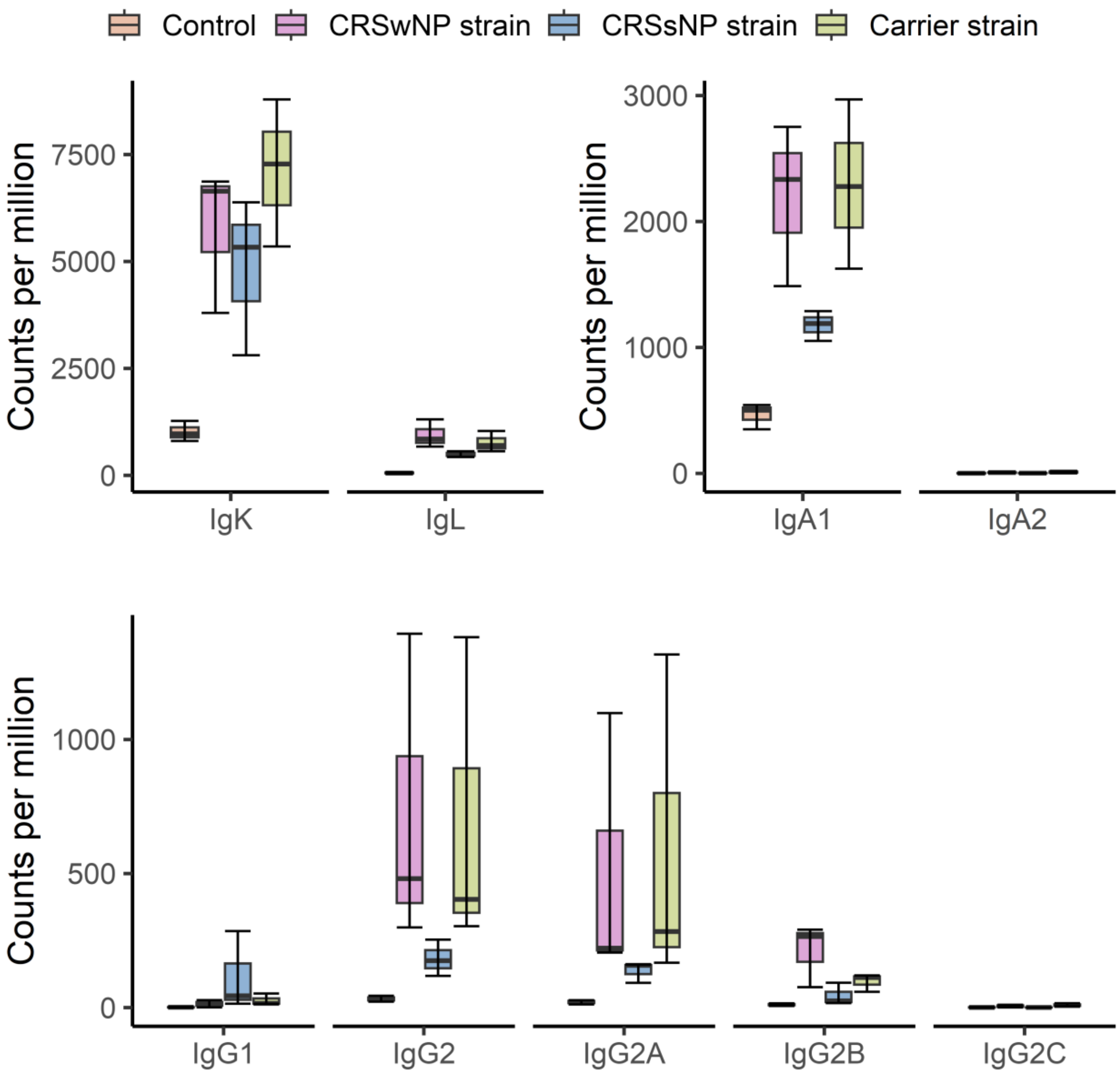

Figure S1. Nasal mucosal tissue transcriptome analysis revealed a differential expression of immunoglobulin light chain, IgA, and IgG subtypes after chronic exposure to *S. aureus* biofilm-secreted factors (SABSF). The transcript count (per million) of the subclasses is presented.

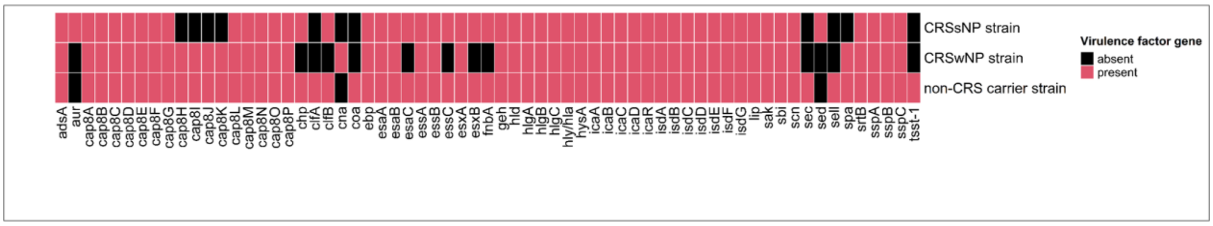

Figure S2. Matrix showing the presence of virulence genes within the genome of clinical isolates identified by Whole-Genome Sequencing and screening against the Virulence Factor Database (VFDB).

Table S1. Quality control parameters transcriptomics

Sample	Group	nanodrop 260/280	nanodrop 260/230	Qubit concentration (ng/µl)	Tapestation RIN	Total read count	Mapped read count	Mean read length (n)	Mean read quality
**sample 1-01**	Control	2.11	2.16	508	7.4	4510776	3387156	967	16.7
**sample 1-02**	Control	2.1	1.87	670	7.1	3863752	3120880	943	15.5
**sample 1-03**	Control	2.09	2.19	810	7.6	2883680	2463364	1005	17.7
**sample 2-02**	CRSwNP strain	2.1	2.26	494	7.9	6301312	5222782	1081	16.4
**sample 2-03**	CRSwNP strain	20.9	2.29	678	7.7	2029628	1789710	1183	17.6
**sample 2-05**	CRSwNP strain	2.07	2.07	1200	7.8	3132568	2623119	848	17.4
**sample 3-02**	CRSsNP strain	2.11	2.29	570	8.8	1747104	1523793	1224	17.5
**sample 3-03**	CRSsNP strain	2.1	2.22	1240	8.6	3252741	2957867	1206	17.5
**sample 3-05**	CRSsNP strain	2.1	1.93	750	9	2331502	2099034	1263	16.9
**sample 4-01**	Carrier strain	2.08	2.21	694	9.1	1384658	1085443	1152	13.8
**sample 4-02**	Carrier strain	2.12	2.16	1640	9.1	3537580	3215375	1190	17.3
**sample 4-05**	Carrier strain	2.08	2.27	694	8.9	893817	789443	1532	19.8

Table S2. Significant differently expressed genes.

See supplementary files

Table S3. Significant gene ontology pathways.

See supplementary files

Table S4. Significant KEGG gene set enrichment pathways.

See supplementary files

Table S5. Significant cell marker pathways.

See supplementary files
