# Supplementary figures and images for: Staphylococcus aureus Biofilm-Secreted Factors Cause Mucosal Damage, Mast Cell Infiltration, and Goblet Cell Hyperplasia in a Rat Rhinosinusitis Model
Source: Int J Mol Sci. 2024 Mar 17;25(6):3402. doi: 10.3390/ijms25063402 (PMC10970533; doi:10.3390/ijms25063402)

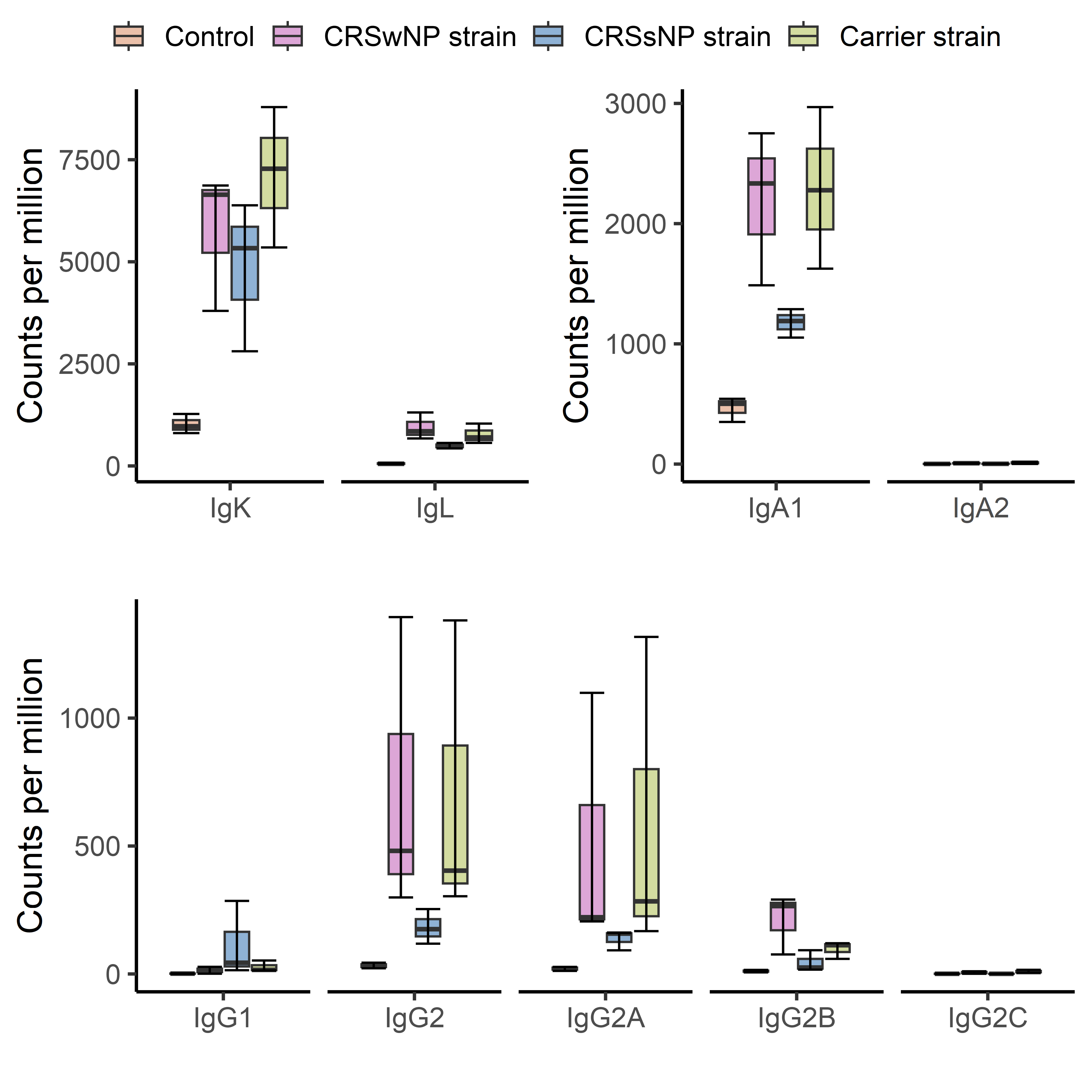

Supplement: Supplementary file 1 [file ijms-25-03402-s001.zip › Figure S1.png]

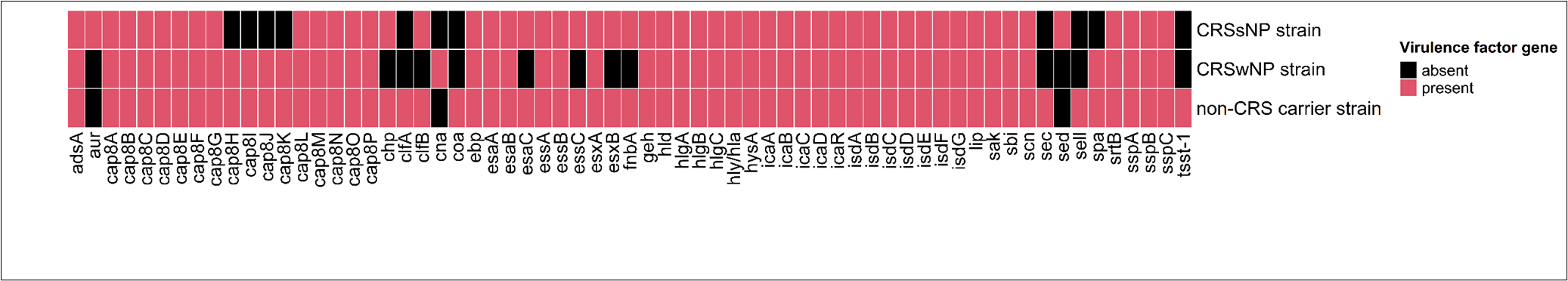

Supplement: Supplementary file 1 [file ijms-25-03402-s001.zip › Figure S2.png]
